# Exploring Cyclodextrin-Based MOFs for Drug Delivery: Synthesis, Applications, and Future Perspectives

**DOI:** 10.1021/acsomega.5c10791

**Published:** 2026-01-22

**Authors:** Şeyma Edisan, N.Başaran Mutlu-Ağardan

**Affiliations:** Department of Pharmaceutical Technology, Faculty of Pharmacy, 37511 Gazi University, Ankara 06630, Türkiye

## Abstract

Three-dimensional (3D) metal–organic frameworks (MOFs), are known by various names, such as organic zeolite analogues, 3D porous coordination polymers, hybrid organic–inorganic materials, coordination polymers, and metal–organic polymers, are advanced three-dimensional materials distinguished by their high surface area, tunable surface properties, and well-defined crystalline structures. Due to these exceptional characteristics, MOFs have been extensively explored for applications in diverse fields, including gas storage, chemical separation, ion exchange, and catalysis. 3D cyclodextrin-based metal–organic frameworks (CD-MOFs) have emerged as a biocompatible alternative to conventional MOFs, as they are synthesized using safer, nontoxic, or lower-toxicity components, thereby eliminating the need for potentially hazardous metals and organic linkers commonly employed in traditional MOF synthesis. In CD-MOFs, cyclodextrin molecules serve as organic linkers, while metal sources, such as KOH, NaOH, and KCl, provide the necessary metal ions for framework formation. CD-MOFs form body-centered cubic structures by binding to one of the alkali metal cations through coordination of the secondary face hydroxyl groups on the alternate d-glucopyranosyl residues. Beyond the intrinsic advantages of traditional MOFs, CD-MOFs offer additional benefits, particularly in drug delivery applications, where biocompatibility is a crucial factor. These CD-MOFs can be synthesized through various techniques, and multiple strategies can be employed for drug loading. This review comprehensively examines the synthesis of 3D CD-MOFs, their drug loading methodologies, comparative analysis of these methods in terms of advantages and limitations, and the potential of 3D CD-MOFs as drug delivery systems.

## Graphical abstract

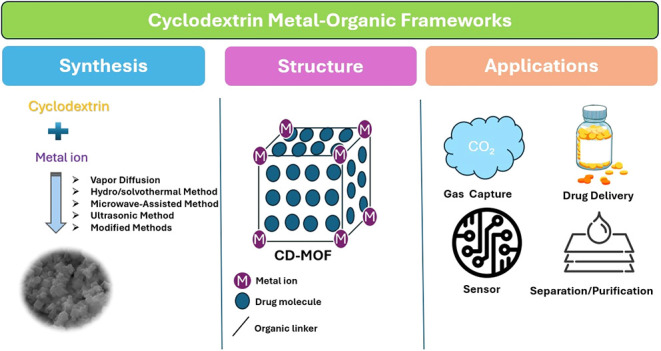

## 1 Introduction

Porous materials constitute a versatile class of materials widely used for drug delivery purposes offering advantages due to their large surface area and large pore volumes such as drug loading, control of drug release rate, modification of the dissolution rate of poorly soluble drugs, and protection of the drug from external conditions. The large surface area can contribute to the amorphization of the drug, thereby preventing its crystallization. The adsorption potential of porous materials of external loads also provides application areas in processes based on adsorption phenomena, such as gas drying, the synthesis of supported catalysts, and separation processes.^1^

According to The International Union of Pure and Applied Chemistry (IUPAC), porous materials could be classified according to their pore sizes into three main categories: micropores (<2 nm), mesopores (2–50 nm), and macropores (>50 nm). In this concept, MOFs with high porosity and surface area can be classified as microporous MOFs and mesoporous MOFs. Microporous MOFs are used to accommodate small molecules, while mesoporous MOFs are used to incorporate larger molecules and have been studied in recent years as excellent carriers for drug delivery.^2^

MOFs consist of metal ions and organic ligands used as linkers.^3^ A lattice-shaped crystal structure is formed by the coordination of metal ions with organic ligands ­(Figure 2). Metal–organic frameworks (MOFs) are categorized as one-dimensional (1D), two-dimensional (2D), and three-dimensional (3D) coordination networks, depending on the spatial connectivity of the metal ions and organic linkers (Figure 1). This dimensionality profoundly affects their physicochemical properties, such as porosity, surface area, diffusion behavior, and mechanical stability. In 1D CD-MOFs, metal ions coordinate with cyclodextrin (CD) units to form chain-like structures. These structures exhibit limited porosity but provide well-defined channels for guest molecule transport. 2D CD-MOFs form layered sheet-like structures connected in planar arrangements by metal ions. Interlayer spacing and hydrogen bonding interactions determine their flexibility and guest adsorption properties. Recent studies suggest that 2D frameworks can enhance diffusion kinetics and provide tunable surface functionality for drug loading. 3D CD-MOFs, typically constructed from γ-cyclodextrin and alkali metal ions, have a highly porous cubic structure with interconnected cavities. Their large surface area and uniform pore distribution enable the efficient encapsulation of a wide variety of drug molecules. The three-dimensional connectivity also enhances framework stability.^4^

**Figure 1:**
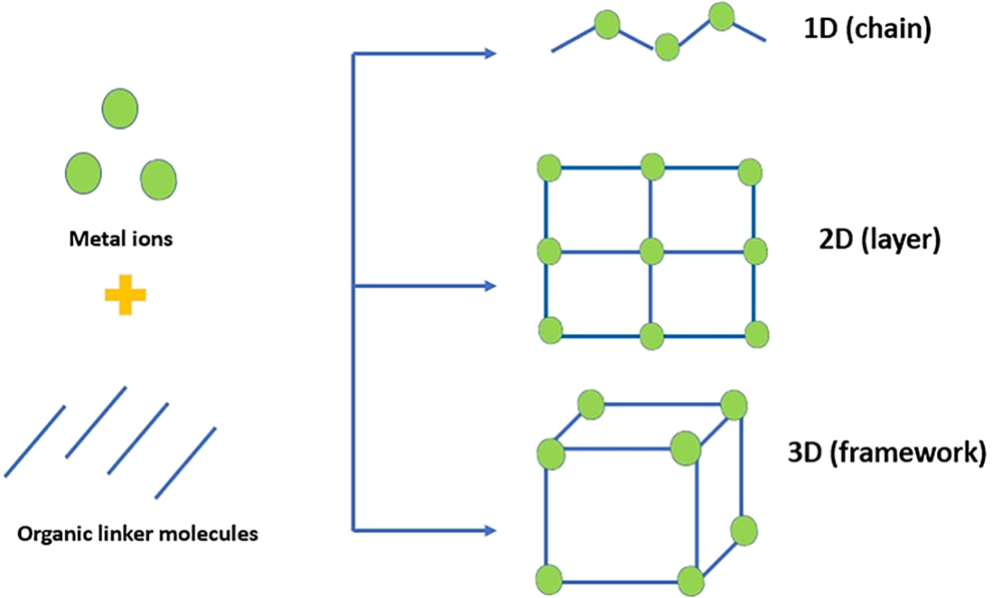
One-dimensional (1D), two-dimensional (2D), and three-dimensional (3D) coordination networks.

**Figure 2:**
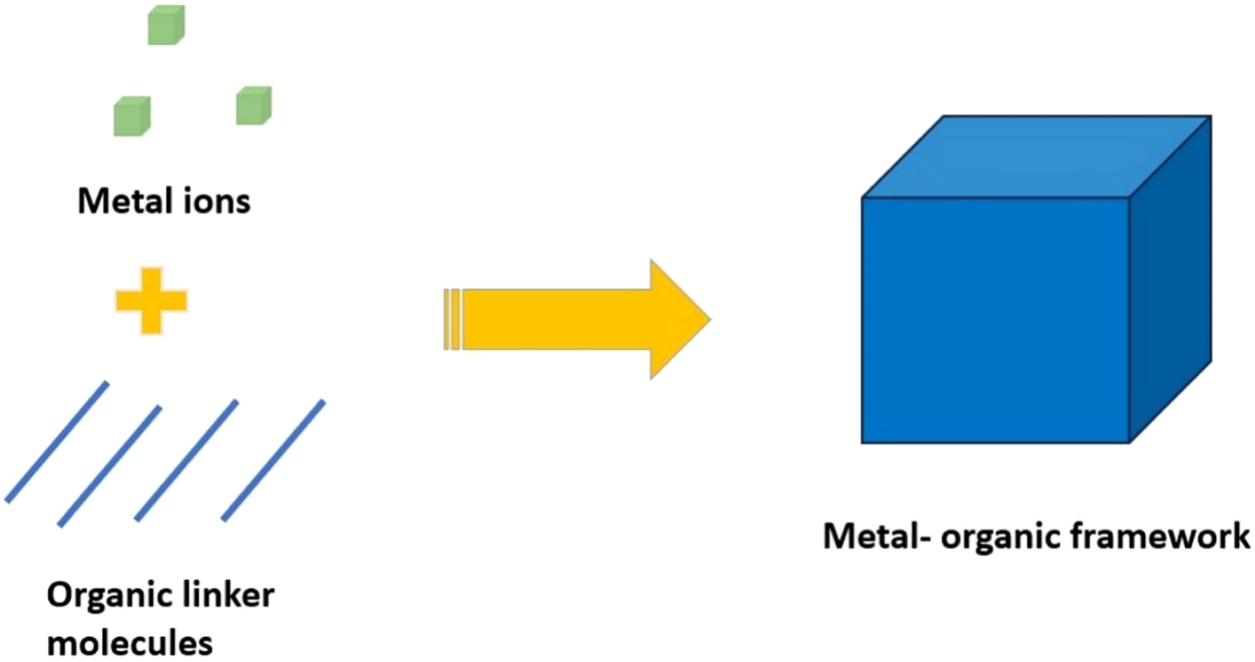
Structure 3D of the metal–organic framework.

MOFs could be prepared using a wide variety of metal ions such as Fe, Zn, Mg, Rb, and Cs.^5^ The most common organic linkers are sulfonate, carboxylate, or phosphonate structures.^5^

MOFs have been investigated for many purposes such as gas storage,^6^ chemical separation,^7^ catalysis,^8^ ion exchange,^9^ drug delivery,^10^ and adsorption^11^ (Figure 3 *)*. Compared to conventional porous materials, MOFs have recently attracted attention due to their large surface area, adjustable pore size, simple synthesis methods, high porosity, functional surface chemistry, and tunable internal surface properties.^12^ However, toxicity remains a concern due to the presence of metals and organic linkers that constitute the framework.^13^

**Figure 3:**
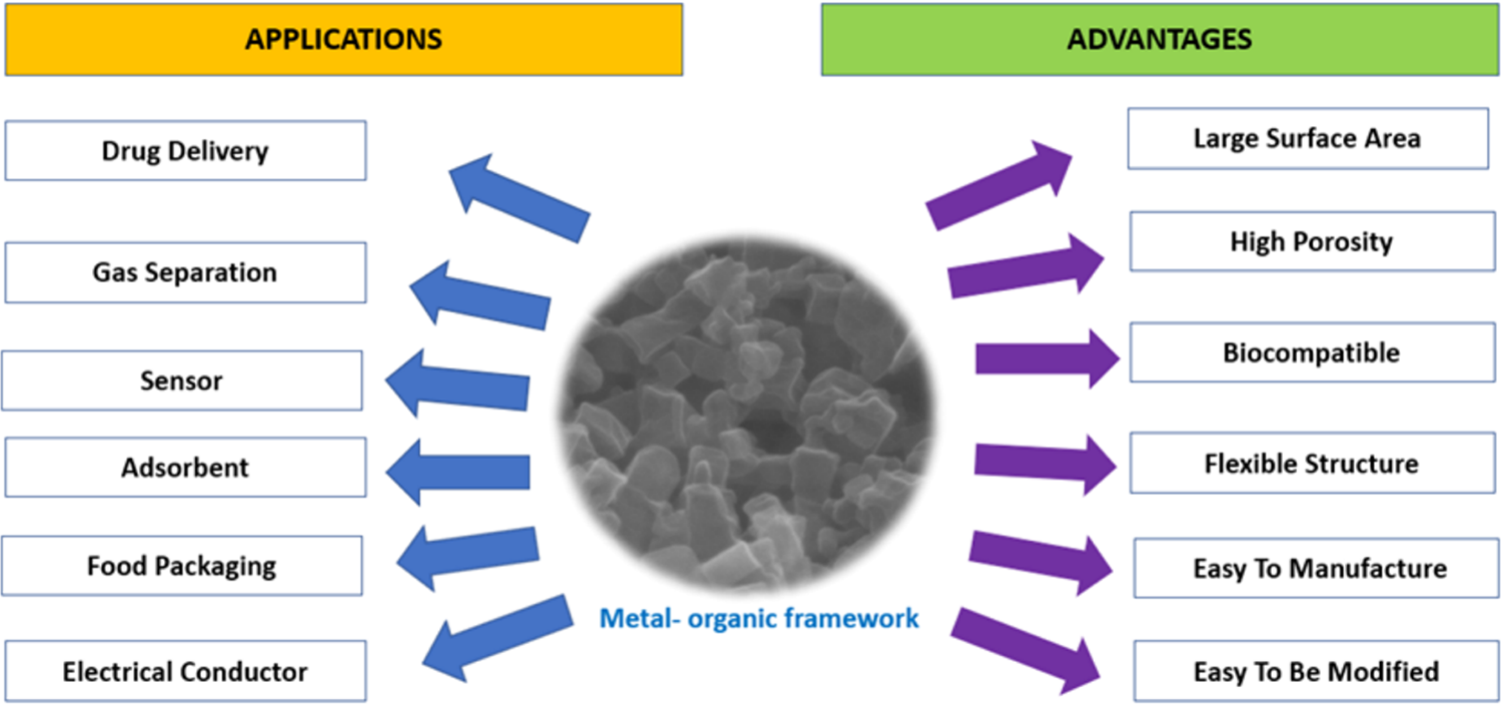
Advantages and applications of metal–organic frameworks.

To mitigate toxicity concerns, MOF research has increasingly focused on the synthesis of MOFs using nontoxic metal ions (such as calcium, potassium, sodium, and lithium) and biocompatible materials, including cyclic oligosaccharides, peptides, and carbohydrates. By this concept, cyclodextrins have gained significant attention for the synthesis of metal–organic frameworks over the past decade.

Cyclodextrins (CDs) are cyclic oligosaccharides composed of α-1,4-linked d-glucopyranoside units obtained by the enzymatic degradation of starch. They are truncated cone-shaped macromolecules with a hydrophobic inner cavity and a hydrophilic surface. Owing to the hydroxyl groups, their outer surfaces are hydrophilic, while the glycosidic oxygens and C–H bonds in their inner cavities impart hydrophobic properties to the structure.^14^ There are 3 types of natural cyclodextrins: α-, β-, and γ-CDs, which are composed of 6, 7, and 8 glucose units, respectively, exhibiting different properties such as ring and cavity sizes and solubility properties (Table 1).^15^

**Table 1:** Characteristics of Natural Cyclodextrins

type of CD	cavity diameter (Å)	inner diameter (nm)	molecular weight (g/mol)	solubility (g/100 mL)
α- CD	4.7–5.3	0.57	972	14.5
β-CD	6.0–6.5	0.78	1135	1.85
γ-CD	7.5–8.3	0.95	1297	23.2

As a unique feature, cyclodextrins have the ability to form noncovalent inclusion complexes with metal ions through electrostatic interactions, van der Waals interactions, and hydrogen bonds.^3^ Due to its structure and shape, the cyclodextrin molecule can trap guest molecules in its inner cavity. This feature is utilized in pharmaceutical applications to increase the solubility of active pharmaceutical agents with low water solubility and stability. In addition, they are also used in overcoming many problems such as masking the bad taste of active substances and improving their bioavailability. There are a considerable number of drugs on the market containing cyclodextrin derivatives.^16–18^

Hydrophilic cyclodextrins are nontoxic, available for almost all drug application routes, and accepted as GRAS excipients. Hereby, these properties provide an advantage in overcoming and eliminating toxicity concerns in the preparation of MOFs, suggesting a new platform of MOFs. With this point of view, CD-MOFs synthesized using different cyclodextrin derivatives have been investigated in recent years.

Among three types of natural CDs, MOFs preparation can be achieved most commonly using γ-CD due to the presence of -OCCO- binding groups on the primary and secondary faces, which could be used to complex with metal ions. γ-CD has a larger inner cavity, hydrophobic voids, better bioavailability, and higher water solubility compared to α-CD and β-CD. Due to these advantages, γ-cyclodextrins are more commonly preferred over other cyclodextrin derivatives in studies involving CD-MOFs in the literature.^19–23^

Biocompatible and biodegradable CD-MOFs can be prepared with high solubility, surface area, and porosity properties by taking advantage of both CDs and MOFs. As discussed in this review, 3D CD-MOFs offer many advantages as drug delivery systems. 3D CD-MOFs offer unique advantages such as high surface area, porosity, controllable surface, increased solubility, and high biocompatibility. In the pharmaceutical field, 3D CD-MOFs suggest a safe micro- or nanosized drug delivery system by increasing the solubility of drugs with low solubility and improving their bioavailability, as well as providing controlled release of drugs and suitable for scale-up.^24^ This review provides a brief summary of the synthesis of 3D CD-MOFs and drug encapsulation methods by classifying literature via disease and/or drugs.

## 2 Synthesis Methods of CD-MOFs

Synthesis of CD-MOFs is briefly based on crystal formation by nucleation, which is followed by crystal growth. Precursors dissolved in solution began to precipitate and formed tiny crystals. Growth units are collected on the surface of the formed crystal nuclei and are obtained as crystals. The solvent, commonly preferred as methanol, in the medium accelerates the nucleation process by increasing the saturation of the cyclodextrins.^25^ Various methods have been employed in the synthesis of the CD-MOF structure. It is crucial to select the optimum method and optimize process parameters that directly affect the characteristics of the CD-MOFs, such as particle size, pore volume, and surface area.^26^ This section discusses the various methods employed for synthesis along with their advantages and disadvantages.

### 2.1 Vapor Diffusion Method

The vapor diffusion method is the first and major approach for synthesizing CD-MOFs, based on the principle of liquid–liquid diffusion.^26^ In this method, the solvents form two distinct layers depending on their densities with the first layer containing the precipitating solvent and the second layer containing the product. This method requires 2–7 days at room conditions, which is a relatively long period of time, thus could be considered a time-consuming method.^27^

KOH is the most common excipient used in the CD-MOF synthesis as a metal ion resource. γ-CD-MOFs’ most common synthesis method involves combining 1.0 equiv of γ-CD with 8.0 equiv of KOH in an aqueous solution, followed by the vapor diffusion of methanol (MeOH) into the solution over a period of 2 to 7 days (Figure 4). Smaldone et al. conducted a comparative study by synthesizing γ-CD-MOFs via the vapor diffusion method with different metal ions than K^+^ such as Rb^+^ and Cs^+^.^28^ In another study, Li/K-γ-CD-MOF was synthesized using various ratios of γ-cyclodextrin and metal ions, employing a mixture of Li^+^ and K^+^ ions. CD-MOFs were obtained by dissolving γ-CD, KOH, or KOH/LiOH·H_2_O in distilled water, followed by methanol vapor diffusion for approximately 15 days. The study demonstrated that, in addition to metal ions such as K and Cs, γ-CD-MOFs could also be synthesized by combining Li and K ions.^29^ A group of researchers synthesized CD-MOFs using the methanol vapor diffusion method, with a γ-cyclodextrin to KOH ratio of 1:8 mequiv. Scanning electron microscopy (SEM) analysis revealed cubic, crystalline structures of γ-CD-MOFs, with particle sizes ranging from 40 to 500 μm.^28^ It has been reported that particle sizes vary between approximately 200 nm and 400 μm by employing different molar ratios, metal salts, and diffusion conditions applied in the vapor diffusion method for CD-MOF synthesis.^27^ For example, γ-CD-MOF crystals were synthesized with a particle size of 8.60 ± 1.95 μm under optimized conditions.^30^ These findings emphasize that careful adjustment of the metal/ligand molar ratio, temperature, and diffusion time is necessary to control the final particle size of CD-MOFs.

**Figure 4:**
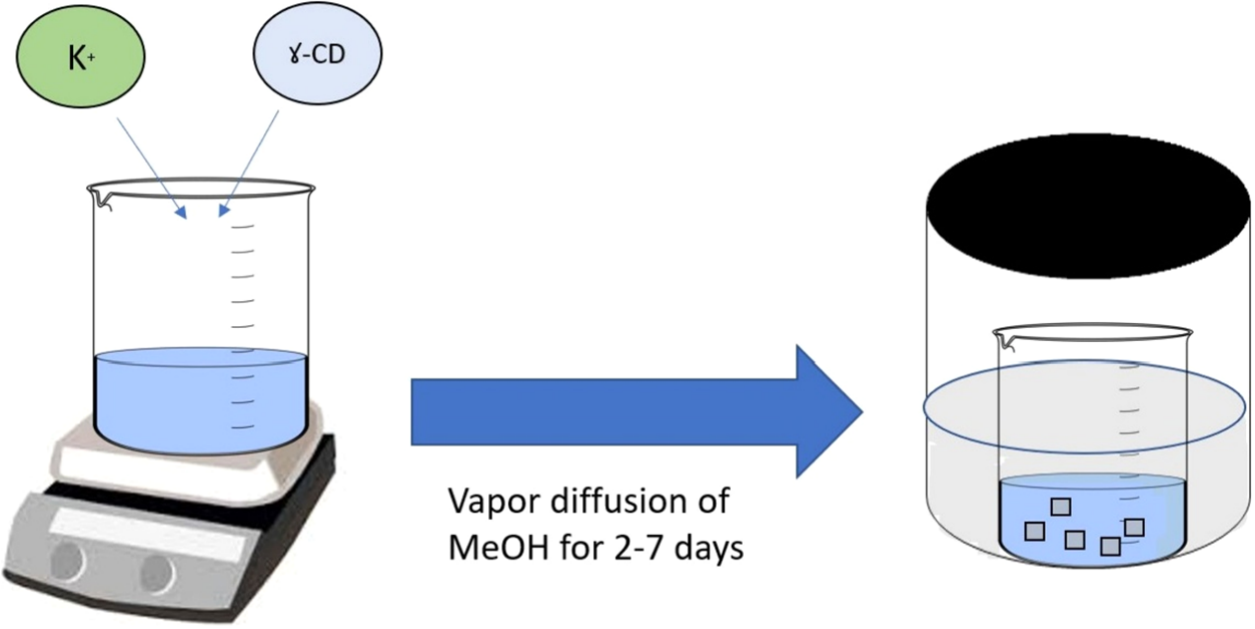
Schematic representation of γ-CD-MOF synthesis via the vapor diffusion method.

### 2.2 Hydro/Solvothermal Method

The Hydro/solvothermal method is carried out in pressure-resistant vessels at high pressure and high temperatures in the presence of solvents such as water and/or organic solvents.^26^ When water is used as the solvent of the reaction medium, the method is called hydrothermal method (Figure 5), while it is called solvothermal method when nonaqueous solvent is used.^27^ The morphologies and particle sizes of CD-MOFs synthesized via hydrothermal and solvothermal methods vary depending on various synthesis parameters such as temperature, reaction time, precursor concentration, and pH. In a study, synthesis performed at 100–120 °C for 6–12 h, generally yielded micron-sized (1–10 μm) γ-CD-MOF particles, while reactions at lower temperatures (70–90 °C) or for shorter times (<4 h) resulted in nanosized crystals in the 500–700 nm range.^27,31^ Increasing the precursor concentration or adjusting the γ-cyclodextrin/metal ion molar ratio increases the nucleation density, contributing to the formation of smaller and more homogeneous crystal sizes. Similarly, pH conditions influence the framework growth kinetics and final particle morphology by regulating the coordination strength between cyclodextrin hydroxyl groups and metal cations. Therefore, systematic optimization of these parameters is crucial for properly controlling CD-MOF particle sizes for targeted drug delivery applications.

**Figure 5:**
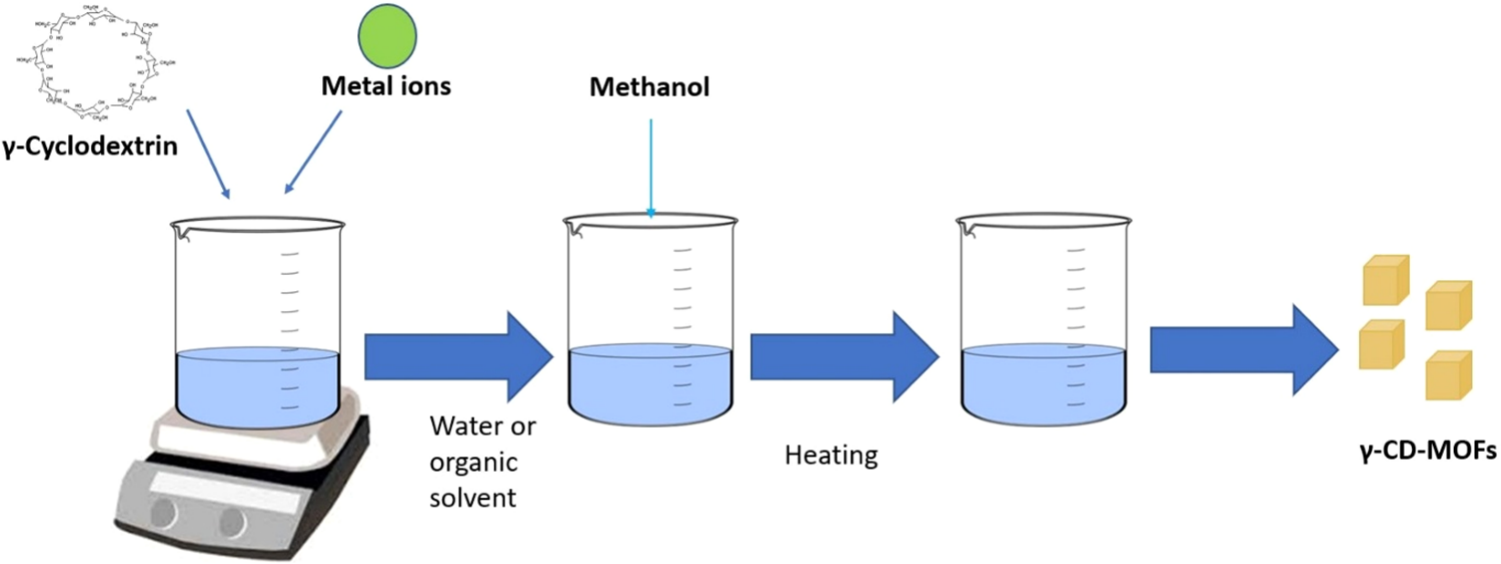
Schematic representation of γ-CD-MOF synthesis via the hydrothermal method.

These parameters were investigated by Hu et al. to evaluate the effects on the size and degree of crystallization of γ-CD-MOFs. As a result, micrometer (5–10 μm) and nanometer (500–700 nm) sized γ-CD-MOFs were obtained by combining KOH and γ-CD in an aqueous solution and adding methanol for 6 h at 50 °C^32^ suggested as optimum conditions. In a study in the literature, β-CD and Na_2_C_2_O_4_ were added to a solution mixture consisting of methanol and water and heated at 160 °C for 3 days, and β-CD-MOFs were prepared by this method and suggested as a potential drug delivery system by loading 5-FU.^33^ In another study, γ-CD-MOFs were successfully synthesized by using the hydro/solvothermal method. A mixture of γ-cyclodextrin (324 mg), potassium hydroxide (8 mg), and deionized water (10 mL) was combined with 12 mL of methanol. The solution was then heated at 50 °C for 20 min and subsequently centrifuged. Micron-sized γ-CD-MOFs were collected and washed twice with 15 mL of ethanol and methanol. Finally, the γ-CD-MOF crystals were dried overnight at 50 °C under vacuum. The surface morphology of the synthesized γ-CD-MOFs revealed uniform cubic crystals.^34^

### 2.3 Microwave-Assisted Method

The microwave-assisted method has been successfully employed for the synthesis of various materials, metal oxides, inorganic hybrids, and MOFs. This time-saving technique is characterized by its simplicity and cost-effectiveness. In addition to being relatively environmentally friendly and efficient, it also provides advantages such as rapid heating and high energy efficiency.^26^ Compared with the traditional heating method, microwave synthesis is characterized by the energy transfer occurring within 1 ns through direct heating in the reaction medium, providing instant heating. In the process of CD-MOF synthesis, the advantage of this technique is that it can evenly increase the temperature of the whole sample, especially by making the temperature of the center of the reaction mixture the same as the temperature of the edge of the reaction mixture, which leads to crystal nucleation and growth. Although the microwave synthesis method seems advantageous due to its low energy consumption, rapid and cost-effective nature, and being a green method, providing the same conditions with different devices may hinder the reproducibility of the product.^35,36^

Microwave-assisted synthesis was successfully employed to prepare micro- and nanometer-sized γ-CD-MOFs. γ-CD-MOFs were synthesized by using γ-CD and KOH in a molar ratio of 1:8. Cubic γ-CD-MOF crystals were obtained under varying reaction conditions. Specifically, 324 mg of γ-cyclodextrin and 112 mg of potassium hydroxide were dissolved in 10 mL of water, followed by the addition of 6 mL of methanol (Figure 6). The mixture was then subjected to microwave irradiation at temperatures ranging from 10 to 100 °C, with a power of 100 W and durations varying from 1 to 120 min, resulting in a clear solution. To induce crystal formation, 256 mg of polyethylene glycol 20,000 (PEG 20000) was added to the solution. The resulting crystals were washed twice with ethanol and methanol, yielding micrometer-sized γ-CD-MOF crystals. Elaborately, the size and morphology of γ-CD-MOF crystals were investigated by modifying parameters such as the reaction time, temperature, and solvent ratio. Additionally, PEG 20000 and/or methanol were used to obtain nanometer-sized crystals. Notably, nanometer-sized γ-CD-MOFs exhibited a significantly fast and higher adsorption capacity for fenbufen within just a day compared to their micron-sized counterparts synthesized with other methods.^25^ Microwave-assisted synthesis provides rapid and uniform heating, significantly accelerating the nucleation process and providing better control over the particle size distribution. In one study, γ-CD-MOF crystals synthesized at 100 °C for 10 min produced particles with an average diameter of 600–800 nm, while extending the reaction to 30 min resulted in more clustered particles.^37^ Xu et al. observed that solvent polarity affects the morphology of CD-MOFs, with ethanol-rich environments producing more uniform cubic crystals compared to water-dominated systems. These findings collectively highlight that optimizing the temperature, reaction time, and precursor concentration in microwave-assisted synthesis is important to obtain desired nanometer-scale CD-MOFs with enhanced drug loading capacity and cellular uptake potential.^25^ Jia et al. synthesized a γ-CD-MOFscaffold by a microwave-assisted method and integrated graphene quantum dots (GQDs) into its structure to gain a strong fluorescence. By modifying its surface with a pH-sensitive PEGMA polymer and functionalizing it with an AS1411 aptamer, a carrier system capable of targeted and controlled drug release was developed. This system, loaded with doxorubicin hydrochloride, exhibited effective tumor targeting in addition to potent antitumor activity in both in vitro and in vivo studies.^38^ Singh et al. developed γ-CD-MOF and cross-linked γ-CD-MOF (CL-γ-CD-MOF) by the microwave-assisted synthesis method and then aimed to increase cell interaction by surface modification with hyaluronic acid (HA). HA-functionalized γ-CD-MOFs (γ-CD-MOF-HA) showed a 4.8% higher drug loading capacity compared to standard γ-CD-MOFs. Moreover, pH-sensitive drug release and enhanced doxorubicin uptake in HeLa cells were obtained. The results demonstrated the potential of γ-CD-MOF-HA systems as targeted drug carriers.^39^

**Figure 6:**
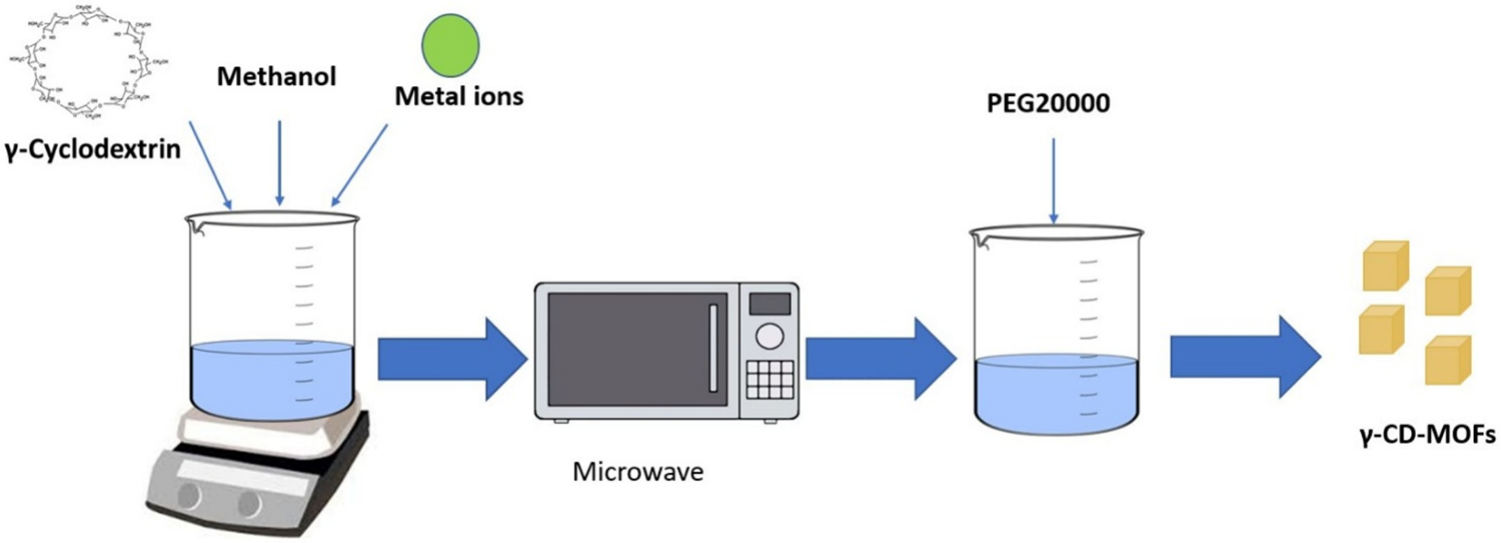
Schematic representation of CD-MOF synthesis via the microwave-assisted method.

### 2.4 Ultrasonic Method

Microwave-assisted method conditions require heating to high temperatures. Therefore, the ultrasound technique is suggested as an alternative time-efficient method using ultrasound to synthesize CD-MOFs. This method requires optimization of the ultrasound power, reaction time, and reaction temperature.^40^

Shen et al. prepared MOFs based on γ-cyclodextrin by ultrasound-assisted rapid synthesis technique (Figure 7). Briefly, 648 mg of γ-cyclodextrin and 256 mg of potassium hydroxide were mixed in 20 mL of ultrapure water and filtered through a 0.45-μm filter, and methanol was added. The clear and transparent solution was ultrasonically processed using an ultrasonic probe at a frequency of 20 kHz and a power of 540 W. Then, 256 mg of PEG 8000 was added to induce the formation of crystals. The morphology of the CD-MOFs obtained under 540 W ultrasonic power was reported to be uniform and cubic shaped, with a 8 μm size.^30^ Studies have shown that the ultrasonic method can be successfully utilized for CD-MOF synthesis. The morphology and size of CD-MOFs prepared with ultrasound-assisted synthesis are homogeneous, cubical, and micron-sized. According to Zhao et al., γ-CD-MOF nanoparticles with an average size of approximately 393 nm were synthesized using an ultrasonic frequency of 40 kHz, power of 240 W, at 25 °C for 30 min in ethanol–water mixture. These parameters promote efficient nucleation and prevent aggregation by providing a uniform acoustic cavitation. By combining the ultrasonic method with ester bond cross-linking strategy, significant improvement was obtained in the water stability of CD-MOF, resulting in a retention of over 90% in various media, while simultaneously facilitating the controlled release of quercetin and demonstrating outstanding antioxidant properties with a free radical scavenging rate of 82.27%.^41^

**Figure 7:**
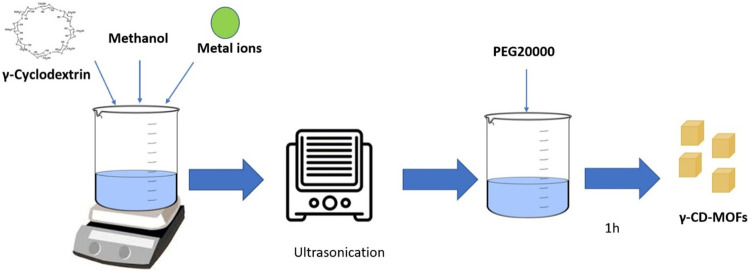
Schematic representation of CD-MOF synthesis via the ultrasonic method.

### 2.5 Spray-Drying Method

Spray drying has recently emerged as a promising alternative to traditional vapor diffusion for the preparation of CD-MOFs. Compared with traditional crystallization techniques, spray drying offers significant advantages, including higher product yields, shorter preparation times, and the ability to control particle size and morphology. During this process, rapid solvent evaporation facilitates accelerated crystal growth, leading to the formation of nanosized MOF particles. On the other hand, the vapor diffusion method relies on a limited amount of organic solvent in the gas phase, resulting in slower nucleation and crystal growth. Therefore, this method lacks the scalability and rapid processing capabilities of spray drying. Importantly, the rapid solvent removal inherent in spray drying can promote the aggregation of nanocrystals and the formation of CD-MOF particles with relatively low crystallinity.^42^ Spray-dried cCD-MOFs with tunable crystallinity, porosity, and dissolution properties have been successfully developed for potential drug delivery applications. Optimization of precursor parameters (ethanol volume fraction, incubation time, and precursor concentration) enabled controlled crystallization. Spray-dried CD-MOFs were categorized as amorphous, partially crystalline, or highly crystalline based on morphology, XRD peak intensity, and surface area. In a study using the spray-drying method, the addition of ketoconazole (KCZ) to the precursor produced KCZ@CD-MOFs with a specific surface area of 292 m^2^/g, which is approximately three times that of conventional CD-MOFs (94.1 m^2^/g). KCZ occupies the hydrophobic spaces between γ-CD molecules, affects crystal growth and dissolution behavior.^42,43^

### 2.6 Modified Methods

In addition to the listed methods, combinatory methods have also been suggested, comprising solvothermal and ultrasonic methods. In this combinatory approach, 3.24 g of γ-CD, 1.12 g of KOH, and 100 mL of water were mixed and subjected to ultrasound treatment for 30 min, followed by filtration. Methanol was then added to the filtrate, and the mixture was heated in an ultrasonic bath until the solution became clear. Crystal formation was induced by adding PEG-20000and MeOH to the clear solution. The resulting crystals were washed with Methanol and ethanol and subsequently left to undergo diffusion in dichloromethane for 3 days. Finally, the crystals were centrifuged and vacuum-dried overnight.^44^ As reported by Kang et al., γ-CD-MOFs were successfully synthesized under solvent-free conditions within 60 min, demonstrating excellent reproducibility and reduced environmental impact. This approach is consistent with green chemistry principles by eliminating the need for toxic organic solvents and high-temperature processing.^45^

Although there are various methods to be used for CD-MOF synthesis, all methods have superiorities and disadvantages, which are presented in Table 2. The optimum method needs to be chosen considering the laboratory facilities, duration of the experiment, and target particle size. In recent years, modified methods have been frequently utilized for CD-MOF synthesis in most of the studies in the literature.

**Table 2:** An Overview of CD-MOF Synthesis Methods

methods	advantages	disadvantages	refs
Hydro/Solvothermal Method	Water or an organic solvent can be used as a solvent.	The method is conducted at ambient pressures exceeding 1 atm and temperatures above 100 °C, in the presence of liquid phases such as water or organic solvents, pressure-resistant apparatuses such as autoclaves or high-pressure reactors are required.	^45,46^
	The method enables the rapid, cost-effective, and efficient synthesis of single crystals in high yields.		
Vapor Diffusion Method	The method requires ambient conditions and pressure.	The ligands used in the method must be soluble. The method is time-consuming (2–7 days)	^47,48^
	This method may be useful for crystallization of proteins.		
Microwave-assisted Method	It is a rapid, simple, and environmentally friendly method.	To provide same conditions with different devices may hinder the reproducibility of the product	^49,50^
	Allows to control crystal distortion.		
	Low-energy-consumption devices are used while generating little chemical waste.		
Ultrasonic Method	The method enables fast, energy-efficient, and room-temperature synthesis.	The ultrasonication step requires an ultrasonic device.	^51,52^
	Green solvents are used instead of toxic organic solvents.	Ultrasound power, reaction time, and reaction temperature should be controlled and optimized.	
	CD-MOFs prepared by this method exhibit enhanced thermal stability.		
Spray-drying Method	The method enables crystal formation in a single step owing to the rapid evaporation of the solvent, therefore offers a fast and scalable production process.	Small changes in feed rate or atomization pressure can significantly affect product morphology and crystallinity, reducing reproducibility.	^42,53^
	It provides energy-efficient and environmentally friendly production enabling less solvent use and high recovery efficiency.	High-temperature use may cause thermal decomposition of cyclodextrin or drug.	
	The method allows precise tuning of particle morphology and size by controlling parameters such as feed rate, temperature, and solvent ratio.		
Modified methods	Traditional methods used in synthesis can be modified. For this purpose, PEG20000 or CTAB is usually used to modify the particle size.	Particle size modulators (i.e., CTAB) used for the synthesis of CD-MOFs may exhibit cytotoxicity; hence, nontoxic and cost-effective surfactants are required	^54–57^
	Modified methods allow nanoscale synthesis in a shorter time.		

## 3 Drug Encapsulation Methods into 3D CD-MOFs

Various drug molecules are encapsulated into CD-MOFs primarily by involving host–guest interactions and the formation of drug nanoclusters. From a thermodynamic standpoint, the most stable conformation is that it possesses the lowest free energy, and this fact establishes the basis of the host–guest interaction mechanism.^58^ Drug molecules preferentially occupy the hydrophobic cavities formed by cyclodextrins (CDs) and ensure the formation of inclusion complexes. The factors contributing to these interactions include the displacement of high-enthalpy water molecules from the CD cavity, hydrogen bonding, van der Waals forces, hydrophobic interactions, and ring strain release. The architecture of CD-MOFs, which combines the hydrophobic cavities of cyclodextrins with a porous coordination network, provides multiple sites for interaction between drug molecules. For example, γ-CD-MOFs-curcumin enhances drug stability and dispersibility by forming hydrogen bonds between the hydroxyl groups of curcumin and the primary hydroxyls of γ-cyclodextrin.^59^ Similarly, β-CD-MOF-ibuprofen complexes exhibit predominant hydrophobic interactions within the CD cavity, facilitating high drug loading and sustained release behavior.^60^ Drug encapsulation into CD-MOFs is facilitated through the diffusion of drug molecules into the cage-like hydrophilic cavities of the framework, typically under high drug concentrations and prolonged loading durations. During the loading of drug molecules into CD-MOF cavities, the limited internal void space leads to uneven distribution of drug molecules, which restricts further drug loading after a period.

In addition to noncovalent interactions, the pore size, surface area, and functional groups of CD-MOFs directly influence the host–guest binding strength and diffusion kinetics. These physicochemical properties govern the adsorption capacity, molecular confinement, and release dynamics. Various analytical techniques are commonly used to characterize these interactions. FTIR spectroscopy is commonly used to detect shifts in characteristic peaks indicative of hydrogen bonding; the PXRD technique to confirm the structural integrity and drug incorporation; BET surface area analysis to measure pore occupancy and adsorption capacity; and DSC and TGA to determine the thermal behavior and confirm inclusion complex formation. Furthermore, molecular docking and computational modeling complement experimental findings are beneficial to provide valuable information to understand binding orientation and energy.^61,62^

Encapsulation of drug molecules into CD-MOF cavities is accomplished using various methods such as cocrystallization, solvent immersion, and mechanical milling. Tamoxifen citrate, valsartan, ibuprofen, azilsartan, and fenbufen are among the drugs encapsulated in CD-MOFs by various methods (Table 3).

**Table 3:** 3D CD-MOFs for Drug Delivery

drug	synthesis method	organic ligand	metal İon	loading method	refs
Ferulic acid	Vapor diffusion	γ-CD	KOH	Cocrystallization	^72^
5-FU	Solvothermal	β-CD	Na_2_C_2_O_4_	Grinding Method	^33^
5-FU	Vapor diffusion	α-CD	KOH	Impregnation	^73^
Captopril/Flurbiprofen	Modified methanol diffusion	γ-CD	KOH	Impregnation	^74^
Fenbufen	Microwave-assisted	γ-CD	KOH	Impregnation	^25^
Lansoprazol	Modified hydrothermal	γ-CD	KOH	Impregnation/Cocrystallization	^31^
Sucralose	Modified hydrothermal	γ-CD	KOH	Impregnation	^75^
Diclofenac sodium	Vapor diffusion	γ-CD	KOH/NaCl/FeCl_3_	Impregnation	^47^
Azilsartan	Solvothermal	γ-CD	KOH	Impregnation	^58^
Budenosid	Modified hydrothermal	γ-CD	KOH	Impregnation	^32^
Valsartan	Hydrothermal	γ-CD	KAc	Impregnation	^76^
Methotrexate	Vapor diffusion	γ-CD	KOH	Impregnation/Cocrystallization	^77^
Ibuprofen	Vapor diffusion	β-CD	KOH	Impregnation/Cocrystallization	^78^
Menthol	Vapor diffusion	α-CD	KNO_3_	Impregnation	^79^
Thymol	Hydrothermal	γ-CD	KOH/KCl/KAc	Cocrystallization	^80^
Tamoxifen citrate	Modified methanol diffusion	γ-CD	KOH	Impregnation	^81^

### 3.1 Cocrystallization

The purpose of cocrystallization is to prepare drug-loaded CD-MOFs by combining drug molecules and CDs with metal ions such as KOH and NaOH, in a similar manner to the synthesis of conventional CD-MOFs. With this method, CD-MOF synthesis and encapsulation are performed in a single step. Cocrystallization offers high encapsulation efficiency with interactions such as hydrogen bonds and van der Waals interactions, as well as the advantages of simple process, low cost, and short time consumption. Due to the presence of high levels of alkali in the synthesis medium, the cocrystallization method is not applicable for alkali-sensitive APIs. The cocrystallization method represents a template-assisted synthesis strategy in which guest molecules actively participate in the formation of the MOF framework. During this process, guest molecules serve as structural templates, guiding crystallization and encapsulating them within the framework. This approach facilitates homogeneous distribution of guest molecules, providing enhanced control over particle morphology.^63,64^

In this method, first, drug, CD, and KOH are dissolved in distilled water, and the obtained solution is filtered through a 0.45 μm organic filter . This is followed by methanol vapor diffusion for 24 h. The synthesized drug-loaded CD-MOF crystals are then separated by filtration, and then the loaded formulations obtained are dried in the oven by washing with ethanol to remove unbound drug molecules from the particle surface.^26^ Hartlieb et al. used the cocrystallization method to obtain ibuprofen-loaded CD-MOFs. CD-MOF formulations obtained with a loading efficiency of 23% increased the solubility of ibuprofen, a widely used nonsteroidal anti-inflammatory drug. Furthermore, in vivo studies have shown that the maximum concentration of ibuprofen in plasma samples is rapidly reached within 10–20 min. The IBU-CD-MOFs has been suggested as an ideal delivery system for analgesic drugs for rapid pain relief.^65^ In another study, CD-MOFs were developed to increase the bioavailability and therapeutic effect of sulfasalazine using two different encapsulation methods for comparison. The loading efficiency of the drug by the impregnation method was 19 wt %, and it was 40 wt % by the cocrystallization method.^66^ Rodríguez-Martínez et al. developed γ-CD-MOFs as a novel drug delivery system for cannabinoids using olivetol (OLV) as a model compound. γ-CD-MOFs were prepared by a microwave-assisted method using different potassium sources such as KOH, KCl, and KNO_3_, and encapsulation of olivetol (OLV) was achieved by impregnation and cocrystallization methods. The encapsulation efficiency was obtained higher by cocrystallization, with γ-CD-MOF-2 (KCl) showing slightly higher OLV content than γ-CD-MOF-3 (KNO_3_), reaching encapsulation from 2% to 10%.^22^

### 3.2 Adsorption (Impregnation)

The impregnation method represents one of the simplest and most versatile approaches for drug loading into CD-MOF. Many studies have applied kinetic and isotherm models to describe the adsorption behavior of drug molecules onto CD-MOFs. For example, pseudo-second-order kinetic models were found to best fit experimental data, while Langmuir isotherms suggested that monolayer adsorption on homogeneous surfaces.^67^ The adsorption capacity of γ-CD-MOFs generally ranges from 60 to 150 mg.g^–1^, depending on pore volume, surface area, and solvent polarity. These findings highlight that the physicochemical properties of CD-MOFs, such as surface functionality, play a critical role in determining the adsorption efficiency and release kinetics.^68^

The adsorption encapsulation method is widely employed for drug loading into CD-MOFs, requiring precise control over parameters such as the mass ratio between drug and CD-MOFs, encapsulation duration, and temperature. This method consists of three main steps: (i) CD-MOF synthesis, (ii) activation of MOFs by removing solvents and/or ligands from the CD-MOF pores, and (iii) loading of the API into CD-MOFs using suitable solvents. The solvent choice is also a critical factor that significantly affects the encapsulation efficiency.^26^

Hartlieb et al. reported that the use of ethanol as a solvent increased the loading of ibuprofen into CD-MOFs to 26% by weight, which was attributed to an anion exchange process wherein ibuprofen was deprotonated by hydroxyl groups in the CD-MOF structure, leading to the formation of an anion that stabilized the positive charge of the remaining framework.^60^ Briefly, in this method, a suspension is obtained by dispersing dry CD-MOF crystals in a drug solution prepared in an organic solvent, followed by incubation at 20–30 °C for a predetermined time period under continuous stirring. The drug-loaded CD-MOFs are then collected by filtration or centrifugation, washed with a solvent such as ethanol, methanol, or IPA to remove unencapsulated drug, and subsequently dried under vacuum.

He et al. used the supercritical carbon dioxide impregnation technique to encapsulate Honokiol (HNK) into γ-CD-K-MOFs as a model drug with low water solubility. This approach improved the water solubility, oral absorption, and bioavailability of HNK. Using the impregnation method, the γ-CD-K-MOFs were dispersed in an ethanol solution of HNK to obtain γ-HNK@CD-K-MOF. The γ-CD-K-MOF activated by supercritical carbon dioxide had a higher drug loading rate.^69^ In the study conducted by Kritskiy et al. on leflunomide (LEF), the aim was to increase the solubility of leflunomide and LEF was encapsulated into γ-CD-K-MOFs by impregnation and cocrystallization methods. The formulations prepared by two different methods increased the solubility of LEF by 80-fold and 30-fold in pH 7.4 buffer for impregnation and cocrystallization, respectively.^70^ Liu et al. developed FEN-γ-CD-MOFs by successfully encapsulating fenbufen (FBF), an analgesic with low water solubility, adding γ-CD-MOFs to an ethanol solution of FBF and reacting them for 24 h until a high loading of FBF up to 196 mg/g was obtained.^25^

### 3.3 Grinding

Grinding is triggered by mechanical forces generated during the mixing of solid components in a mortar and pestle. Guest molecules can be encapsulated into CD-MOFs through mechanical milling, a solvent-assisted technique that facilitates host–guest interactions. In this method, guest molecules and CD-MOFs are weighed in a certain stoichiometric ratio and ground for a specific duration at a controlled temperature by using appropriate solvents. The resulting products are then rinsed with a suitable solvent and dried to a constant weight. Additionally, key factors such as the stoichiometric ratio, working temperature, and grinding duration significantly affect the encapsulation and overall inclusion efficiency.^26^ β-CD-MOFs were synthesized via a grinding method using 5-fluorouracil (5-FU) as a model drug, and their inclusion efficiency into the CD-MOF was investigated at room temperature. The results demonstrated that the inclusion rate of 5-FU in the CD-MOF was 23.02% when the molar ratio of 5-FU-CD-MOF-Na was kept at 1:1. This encapsulation efficiency was notably higher than that of the native β-CD drug complex, which exhibited an inclusion efficiency of 15.73%.^33^ Inoue et al. aimed to expand the clinical applications of ursolic acid, which has a low solubility and bioavailability, by increasing its solubility. For this purpose, CD-MOFs were prepared, and ursolic acid was encapsulated by the grinding method. When the results obtained were analyzed, it was seen that the solubility of the developed formulation was improved 3871 times compared to pure ursolic acid.^71^

## 4 3D CD-MOFs as Drug Delivery Systems

Porous materials are widely used in many fields due to their large surface area and rapid diffusion of electrons through the pores. Porous materials include polymer foams, activated carbon, porous metals, and zeolites. Recently, MOFs, which are porous and have a large surface area, are becoming popular. MOFs are nanoporous structures formed by the bonding of metal ions/clusters and organic ligands to form one-dimensional (1D), two-dimensional (2D), and three-dimensional (3D) structures. Transition metals, alkaline earth metals, and mixed metals are used as inorganic metals, while carboxylates, sulfates, phosphonates, azoles, and heterocyclic compounds are widely used as organic linkers for MOF preparation. Having a large surface area and pore structure, MOFs have exhibited a superior capacity to carry larger amounts of molecules/solvents compared to other porous materials. Another advantage of MOFs is their ability to change the composition of metal–organic ligands, hence exhibiting various properties and controlling the size of the pores. For all these reasons, MOFs are used in a wide variety of applications such as hydrogen storage and separation, catalysis, and drug delivery.^82^ In the past decade, MOFs have gained attention in biomedicine and pharmaceutical research due to their large surface area, tunable pore size, and internal surface properties. The use of conventional MOFs for therapeutic applications is limited due to the toxic chemicals essential for their synthesis. The toxicity originates from the metal ions such as cobalt­(II), cadmium­(II) in the traditional MOF structure and the phosphonate, sulfonate, phenolate structures which are used as binders.^13^ Metal ions used in the synthesis of MOFs exhibit their inherent toxicity when accumulated in the body. Therefore, the concentration of metal ions in MOFs should be kept within the limits allowed for biological use. To avoid toxicity, it is necessary to select metal ions with higher limits allowed for daily exposure of humans. Mg, Ca, Fe, and Zn are some of the metals considered safe for drug release and theranostic applications with established toxicity profiles.^83^ The organic part of the MOFs should also be biocompatible. Biologically acceptable linkers such as peptides, carbohydrates, amino acids, and CD derivatives have been used to minimize the risk of toxicity associated with MOFs. CDs are safe cyclic oligosaccharides commonly used in metal–organic frameworks. To overcome the toxicity problems of conventional MOFs, 3D CD-MOFs are prepared by using different CDs as organic binders due to their safety and biocompatibility.^3^

Research on the use of 3D CD-MOFs as drug delivery systems has increased in recent years. In the literature, studies have been conducted on the development and research of 3D CD-MOF formulations by loading different APIs such as methotrexate, azilsartan, triptolide, quercetin, diclofenac sodium, ibuprofen, lansoprazole, and doxorubicin. In this review, a detailed review of 3D CD-MOF formulations developed as drug delivery systems has been conducted according to the therapeutic indications of the drugs.

### 4.1 CD-MOFs for Analgesic and Anti-Inflammatory Treatment

Inflammatory response is recognized as an important pathogenesis in various diseases.^84^ The innate immune response protects the host against inflammatory processes and moreover activates the innate inflammatory response system. Anti-inflammatory drugs are crucial in maintaining the balance between the inflammatory and immune responses. The limitation of treatment with conventional anti-inflammatory drugs is the inability of the drug to distinguish between healthy and inflammatory tissue and the increased toxicity due to the high dosage. Recent studies have focused on hybrid materials with anti-inflammatory drug delivery.^85,86^ CD-MOFs have been used to overcome these drawbacks of anti-inflammatory drugs, and some studies have been released on this subject.

Nasal administration offers distinct advantages, such as rapid local effect and avoidance of hepatic first-pass metabolism, making it ideal for anti-inflammatory treatments. In contemporary practice, the nasal route of administration is commonly used in the treatment of localized upper respiratory tract pathologies, such as nasal congestion, infectious rhinitis, and allergic nasal disorders. Mometasone furoate (MF) is a topical corticosteroid used to reduce allergic and inflammatory symptoms. γ-Cyclodextrin metal–organic frameworks (γ-CD-MOFs) were incorporated into the hydrophobic cavities to prepare MF@ γ-CD-MOFs powders for nasal application. Drug loading was optimized by incubating MF with a 40 °C, 1 h incubation at a 4% ratio with γ-CD-MOFs. A transparent biomimetic model of the human nasal cavity was produced using 3D printing and used to evaluate intranasal accumulation patterns. PXRD and FTIR analyses confirmed the successful loading of MF into the γ-CD-MOFs. Using the 3D biomimetic nasal cavity model, it was found that a 30° application angle resulted in reduced drug accumulation in the nasal vestibule and increased accumulation in the respiratory and olfactory regions compared to those with a 45° application. In the nasal cavity model derived from male subjects, approximately 51% of the drug reached the respiratory region, while in the model associated with female subjects, nearly 60% of the drug reached this region. Compared to nasal sprays, nasal powder sprays showed less accumulation in the nasal vestibule and more accumulation in the middle-lower nasal concha. The results indicate the suitability of MF@ γ-CD-MOFs for intranasal application and their potential for use as a nasal powder in the treatment of chronic rhinosinusitis.^87^

Abucafy et al. investigated the encapsulation and controlled release properties of sodium diclofenac (DFNa) by different types of MOFs (γ-KCD, γ-NaCD, and γ-FeCD) that were synthesized using γ-CD and compared for oral drug release. The cumulative percentage of drug release for 24 h was observed to be about 63%, 41%, and 42% for γ-FeCD, γ-KCD, and γ-NaCD, respectively. The difference in the drug release profiles was attributed to the variations in the pore volumes of MOFs, and controlled release of sodium diclofenac was achieved with γ-CD-MOFs.^47^ Hartlieb and co-workers synthesized γ-CD-MOF formulations of ibuprofen, which is characterized by low solubility in water and acidic media. Ibuprofen was encapsulated in γ-CD-MOFs with an efficiency of 26%. According to in vivo study data, the tmax value obtained was around 10–20 min, and IBU-γ-CD-MOFs were suggested to be sufficient for a rapid analgesic effect by oral administration. In addition, the bioavailability of ibuprofen was approximately 2-fold increased by IBU-γ-CD-MOFs compared to pure drug.^60^ In another study conducted with ibuprofen, metal–organic frameworks (K-βCD-MOF) based on β-cyclodextrin (β-CD) and potassium ions were synthesized. The effects of factors such as the type and amount of solvent used, the molar ratio of the reagents, and the temperature on the crystallization process were investigated. The increase in the KOH concentration significantly affected the nucleation step, resulting in rapid crystallization. The loading capacity of ibuprofen (IBU) reached 7.4%, and the K-βCD-MOF structure significantly increased the water solubility of ibuprofen, showing potential in pharmaceutical applications.^78^

Delyagina et al. investigated γ-CD-MOFs loaded with tolfenamic acid (TA), a poorly water-soluble anti-inflammatory drug. Although the solubility of TA was very low at pH 1.6, TA-loaded γ-CD-MOFs significantly increased the dissolution rate of TA. At pH 6.8, the release of TA was found to be higher, which was attributed to the increased solubility due to ionization. These results indicate that TA is encapsulated in γ-CD-MOFs in its molecular form, thus leading to an accelerated release profile.^88^

Interestingly, a novel H_2_O_2_-responsive covalent bonded cyclodextrin scaffold (BCOF) was synthesized by Huang et al., aiming to investigate the development and therapeutic efficacy of CD-MOFs for targeted drug delivery, intended for inflammatory bowel disease (IBD). During ulcerative colitis, numerous immune cells accumulate in the colitis tissues and large amounts of reactive oxygen species (ROS) are produced. As a consequence, mucosal ROS concentrations in the tissue abnormally increase by 10 to 100 times. The findings showed that BCOF had an average hydrodynamic diameter of 269.0 nm and a zeta potential of approximately −27.3 mV, indicating the potential for effective drug delivery. In a mouse model of acute ulcerative colitis, BCOF treatment led to improvement in symptoms, and cell viability reached 99%.^89^

### 4.2 CD-MOFs for Tumor Treatment

Currently, cancer is the leading cause of death globally and requires rapid identification and effective antitumor therapies. Nanotechnology plays a significant role in this field by precisely delivering drugs to target cancer cells, minimizing unwanted side effects.^90^ Many of the conventional drugs used in cancer treatment have low solubility and permeability and require high doses for efficient therapy. This causes serious side effects and the development of multidrug resistance (MDR) in cancer treatment. For this reason, new drug delivery systems are being investigated for efficient cancer therapy. Various studies are being conducted to increase the solubility and permeability of some anticancer drugs by using CD-MOFs.

In a study conducted with the chemotherapy agent methotrexate (MTX), MTX was loaded into the γ-CD-MOFs synthesized by the methanol vapor diffusion method by impregnation and cocrystallization methods. It was shown that the dissolution rates of MTX-loaded γ-CD-MOFs were significantly increased compared to pure MTX in physiological buffers and biocompatible media, and a 12.8-fold increase in AUC was observed by the orally administered MTX-γ-CD-MOFs compared to pure MTX. In addition, the prolongation of the elimination time and the higher *C*_max_ values recorded with MTX-γ-CD-MOFs revealed that these systems have potential as oral MTX carriers.^77^ Li et al. developed CD-MOFs by encapsulating triptolide (TPL), which has a narrow therapeutic index, high toxicity, and low water solubility, into the CD-MOF structure in order to increase its solubility, bioavailability, and antitumor effect. It was confirmed by XRD that TPL was loaded into the CD-MOF structure, and it was determined that its water solubility increased by approximately 9.5-fold compared to pure TPL; moreover, extended TPL release was obtained. In vivo pharmacokinetic and antitumor effect analyses showed that loading TPL to CD-MOFs increased the bioavailability of TPL, suggesting a promising drug delivery system for oral TPL treatment.^91^ Tamoxifen citrate (TMX) is a drug widely used in the treatment of breast cancer. In the study conducted by Mutlu-Agardan et al., the effect of γ-cyclodextrin metal–organic frameworks (γ-CD-MOFs) was examined on the permeability and solubility of TMX. TMX-loaded γ-CD-MOFs were obtained by synthesizing γ-CD-MOFs using two methods. As a result of the oral permeability study conducted using the Caco-2 cell line, it was shown that the developed formulations TMX-γ-CD-MOF-1 and TMX-γ-CD-MOF-2 increased oral permeability by 2.24 and 3.57 times, respectively, compared to pure TMX, underlying the role of particle size of γ-CD-MOFs on permeability.^81^ Doxorubicin (DOX) is a drug widely used in cancer treatment. In order to increase the therapeutic efficacy of doxorubicin, CD-MOFs were synthesized and biofunctionalized with hyaluronic acid (HA) to increase drug delivery to tumor sites. According to the results obtained, it was determined that DOX-loaded HA-modified CD-MOFs exhibited significant cellular uptake and biocompatibility, high drug encapsulation efficiency and pH-sensitive drug release.^39^ Another study with doxorubicin (DOX) aimed to control drug release by increasing the stability of the drug using CD-MOFs. In this research, glutathione (GSH)-responsive cubic gel particles (ssCGP) were synthesized through the cross-linking of CD-MOF templates. The DOX@ssCGP formulation was prepared. Glutathione and tris­(2-chloroethyl) phosphate (TCEP) were used as reducing agents capable of breaking disulfide bonds. The studies showed that DOX@ssCGP released less than 10% of DOX in phosphate-buffered saline (PBS, pH 7.4) over 120 min. In the presence of 100 mM GSH and TCEP, almost complete drug release was recorded, indicating a redox-sensitive release mechanism.^92^

### 4.3 CD-MOFs for Antimicrobial Treatment

Antibiotics are a broad class of drugs widely used to treat bacterial infections which inhibit the growth of bacteria or induce cell death, based on effecting several cellular processes.^93^ Misuse of antibiotics and limitations of conventional antibiotic dosage forms are the main factors contributing to antibiotic resistance. The main factors inducing antibiotic resistance include poor solubility and bioavailability of hydrophobic antibiotics, short systemic circulation times and half-lives, increased exposure of healthy tissues due to the lack of selectivity and targetability, and low cellular uptake. Due to these facts, the use of conventional antibiotic dosage forms often fails to achieve sufficient concentrations at infection sites, necessitating increased antibiotic doses and frequent administration, which are associated with more side effects and low patient compliance. Therefore, all these challenges highlight the demand for effective drug delivery strategies to restore and enhance the activity of antibiotics.^94^ Florfenicol and enrofloxacin antibiotics, used in the treatment of infections caused by Gram-positive and Gram-negative bacteria, were encapsulated into γ-CD-MOFs structure, which was synthesized by an ultrasonic method. In order to increase stability, γ-CD-MOFs were subjected to surface modification by the impregnation method with Pluronic L63 and loading of antibiotics into CD-MOF pores was confirmed by PXRD. The in vitro release studies performed at pH 7.4 suggested that antibiotics were released gradually over 4 h and γ-CD-MOF was determined to be an effective carrier system for controlled antibiotics release.^95^

### 4.4 CD-MOFs for Wound Healing

Chronic wounds such as diabetic ulcers and pressure sores have become a global health problem over the last few decades. Acute or chronic wounds caused by injuries and burns can cause various complications, predisposing patients vulnerable to bacterial infections.^96^ The increasing prevalence of these wounds necessitates searching for effective therapies to improve healing outcomes and overcome possible bacterial infections. Recently, nanomaterials have attracted significant attention in this regard due to their superior properties, which potentially offer certain advantages for use in wound management.^97^ Among the various studies conducted on nano-drug delivery systems such as liposomes, nanoparticles, and nanofibers, notable studies were also carried out investigating the susceptibility of CD-MOFs for wound healing.

Lie et al. developed an injectable and self-healing hydrogel system based on quaternary ammonium chitosan (QCS), oxidized hyaluronic acid (OHA), and K-γ-CD-MOF to support poor wound healing in diabetic patients. K-γ-CD-MOFs synthesized by the methanol vapor diffusion method were loaded with α-lipoic acid (α-LA) by the ultrasound technique, and then the hydrogel formulation was prepared by mixing 2.5% (w/v) OHA and 1.5% (w/v) QCS solution. As a result of release studies, it was determined that α-LA was released by 83.4% from the free hydrogel and 72.4% from the hydrogel containing K-γ-CD-MOF. It was concluded that this hydrogel successfully supported wound healing in in vivo diabetic rat models.^98^ Silver nanoparticles (Ag NPs) are promising for combating bacterial resistance.^99^ In the study, it was aimed to increase the stability of silver nanoparticles (Ag NPs) that exhibit inadequate stability, by combining them into CD-MOFs. Ultrathin Ag NPs of 5–6 nm size were adsorbed to CD-MOF crystals by the reaction-diffusion method and cross-linking process, and moreover, surface modification with GRGDS was carried out. GS5-CL-Ag@CD-MOF formulation decreased the clotting time by 39.5% to 2.9 min, accelerated wound healing by 90% on the 10th day and exhibited noteworthy antibacterial efficacy by completely inhibiting *E. coli* growth in 6 h at 32 μg/mL Ag concentration.^100^

### 4.5 CD-MOFs for Pulmonary Drug Delivery

Pulmonary drug delivery (PDD) offers an important drug delivery route due to its advantages over systemic administration, such as minimizing systemic side effects, increasing drug stability, reducing dose frequency, and improving patient compliance. The development of nanotherapeutics with diverse formulations in pulmonary diseases significantly supports treatment safety by closing important gaps in current therapeutic protocols.^101^ Inhalation therapy also offers benefits for the treatment of lung cancers. Compared to systemic chemotherapy, systemic side effects are reduced as the chemotherapeutic agent reaches the tumor site directly; in addition, the first-pass effect is eliminated.^102^

Ren et al. studied the use of luteolin (LUT) loaded CD-MOFs to treat fibrosing interstitial lung disease (ILD) by inhalation, with the aim of improving drug solubility and absorption. Dry powder inhalers with LUT@CD-MOFs were prepared using the solvent incubation method, and aerosol performance was evaluated. In vivo studies demonstrated that inhaled LUT@CD-MOF significantly improved absorption and bioavailability in rats, obtaining a fine particle fraction of 59.8% and a 4.03-fold increase in AUC (0-t) in addition to a 9.11-fold increase in Cmax compared to oral administration.^103^ In a study conducted by another research group, β-CD-MOF and γ-CD-MOFs were synthesized by the solvothermal method, and D-Limonene (D-Lim) was encapsulated into these structures in order to increase the physical stability of D-Lim and develop an inhalable dosage form, to inhibit lung inflammation. The cumulative distribution of particles below 5 μm in size was found to be 36.51 ± 8.38% for γ-CD-MOF and 41.42 ± 9.40% for D-Lim@γ-CD-MOF. D-Lim@γ-CD-MOF prepared in DPI (dry powder inhaler) form was successfully converted into an inhalable formulation with a a fine particle fraction (FPF) of 33.12 ± 1.50%; in vivo studies revealed a 2.3-fold higher bioavailability compared to oral administration.^104^ CD-MOFs have also been investigated as carriers for cyclosporin A (CsA) to improve its delivery via dry powder inhalers (DPIs). CD-MOFs were selected for their ability to effectively encapsulate CsA while maintaining the crystalline structure, even after drug loading, which is crucial for stability and delivery efficiency. The particle size of CD-MOFs, which is affected by different modulators, plays a significant role in the aerodynamic performance of DPIs, with sizes ranging from 3.88 to 11.70 μm. The findings show that CD-MOFs are safe and biocompatible and do not exhibit cytotoxicity or organ damage, and suggested as encouraging systems for pulmonary drug delivery.^105^ An optimized inhalable dry powder formulation using CD-MOFs was developed to enhance the delivery and efficacy of chemotherapeutic drugs, such as PTX in the treatment of lung cancer. A single-factor Box-Behnken design was used to optimize the formulation focusing on variables such as PTX concentration, stirring time, temperature, and molar ratio of γ-CD-MOF to optimize the synthesis procedure. The final formulation was obtained with a 34 mg/mL PTX loading efficiency with a molar ratio of 1:20. The results show that the γ-CD-MOF-PTX formulation improved the pharmacokinetics of PTX with a higher plasma concentration and enhanced pulmonary deposition, reaching an FPF value of 68.8% at a flow rate of 90 L/min.^106^ Levo-tetrahydropalmatine (L-THP) is an alkaloid with poor solubility and bioavailability used in pulmonary diseases, including acute lung injury (ALI). In order to overcome these problems, CD-MOFs were developed by incorporating L-THP into the CD-MOF structure. The results showed that the specific surface area of CD-MOF decreased after L-THP loading, indicating successful incorporation. The flowability was improved by mixing THPCD-MOFs with lactose, and a FPF of 37.25% was obtained with Sv010-L-THPCD-MOF, which was found to be suitable for inhalation.^107^ Another study aimed to design a biodegradable cross-linked covalent cyclodextrin framework (OC–COF), as a reactive oxygen species (ROS)-responsive drug delivery system for the treatment of advanced ALI. OC–COF particles were synthesized by using CD-MOF as a template and oxalyl chloride (OC) as a cross-linking agent. The cubic OC–COF particles with a size of 2–3 μm showed the ability to remove H_2_O_2_, improve cell viability under oxidative stress, and reduce cell apoptosis. The percentage of apoptotic cells decreased from 33.3% to 4.82% as the concentration of OC–COF increased.^108^

### 4.6 CD-MOFs for High Blood Pressure Treatment

Hypertension is one of the most prevalent chronic diseases worldwide, a leading serious risk factor for stroke and cardiovascular diseases. Since the majority of antihypertensive drugs used in the treatment of hypertension exhibit poor bioavailability related to their limited therapeutic efficacy, there is a need to develop safe and effective alternatives. Accordingly, exploiting the unique chemical, biophysical, safety, and efficacy features of nanopharmaceuticals may represent an innovative and effective therapeutic strategy.^109,110^ Based on this perspective, CD-MOFs have been investigated as a drug delivery system in order to provide a more potent treatment by enhancing the bioavailability and solubility of drugs used for hypertension therapy.

In a research reported by Zhang et al., CD-MOFs were synthesized, and valsartan (VAL) was encapsulated to improve the solubility and bioavailability of VAL. VAL/CD-MOFs increased VAL solubility 39.5-fold in pH 6.8 buffer compared to pure VAL. In vitro dissolution data proved the superiority of VAL/CD-MOF capsules over Diovan capsules (89.4%) with 97.5% release within 1 h. In vivo pharmacokinetic analysis revealed that CD-MOF significantly increased the bioavailability of VAL.^111^ In another study, a CD-MOF formulation was developed to improve the solubility and bioavailability of azilsartan (AZL), an angiotensin II inhibitor. Solubility and dissolution studies were performed in the gastrointestinal pH range. The solubility of AZL/CD-MOF increased 340-fold compared to pure AZL and enhanced dissolution rate was obtained, especially in pH 1.0 and 4.5 buffers. In vivo pharmacokinetic analyses revealed that the bioavailability of AZL/CD-MOF was 9.7-fold higher than pure AZL and 1.5-fold higher than AZL/γ-CD complex.^58^

### 4.7 CD-MOFs for the Delivery of Natural Compounds

Natural compounds are bioactive molecules obtained from various sources, including plants, fungi, and marine organisms. Due to their therapeutic effects in different diseases, these substances have attracted increasing attention in recent years. Natural compounds have been shown to have many therapeutic effects, such as antioxidant, anti-inflammatory, and antitumor effects, making them promising drug candidates. Although natural substances have great therapeutic potential, they also have some disadvantages, such as limited water solubility and bioavailability, that make their clinical use. Therefore, in recent years, different drug delivery systems have been developed to overcome these limitations.^112^

In the research of Rodrguez-Martnez et al., it was aimed to encapsulate olivetol (OLV), an intermediate product of cannabinoid biosynthesis, into γ-CD-MOFs. The synthesis was carried out by microwave-assisted technique using different potassium sources (KOH, KCl, KNO_3_). Impregnation and cocrystallization methods were applied for the loading of OLV into γ-CD-MOFs. Higher loading efficiency was obtained by the cocrystallization method with KCl and KNO_3_. The impact of different potassium sources on the pH and morphology of γ-CD-MOFs was evaluated to explain the underlying reasons for the differences in loading capacity. The loading capacity was found to be higher in the cocrystallization method. SEM and PXRD analyses indicated a triangular morphology and different diffraction patterns when different potassium compounds were used, compared to the typical cubic structures produced with KOH. This was explained as being due to the different pH of the initial solutions.^113^

Quercetin (Que), a natural flavonoid with a low water solubility and bioavailability, was encapsulated into γ-CD-MOFs prepared by a modified methanol diffusion method. It was shown that the solubility of Que-CD-MOFs increased 100-fold compared to pure Que, the free radical scavenging capacity improved, and its cytotoxicity against healthy cells decreased while its activity against tumor cells was maintained. Molecular docking studies have shown that Que molecules are localized in the cavities of γ-CD-MOFs, and it is concluded that CD-MOFs may be promising carriers to overcome the solubility and bioavailability issues of natural components.^114^ In order to overcome the solubility, stability, and bioavailability problems of trans–N-P-coumaroyltyramine (N-p-T-CT), which has a therapeutic effect in nerve signal transmission, γ-CD-MOFs were investigated. γ-CD-MOFs were synthesized by the solvent diffusion method, and N-p-T-CT was encapsulated using impregnation and cocrystallization methods. The obtained results show that NCT@CD-MOF has improved the solubility of Npt-CT, the solubility of NCT@CD-MOF in water is 366 times higher than that of free Npt-CT, and a drug loading capacity of 145.03 μg/mg was obtained.^115^ Curcumin is a natural component with a poor physicochemical stability and low oral bioavailability. It was aimed to develop a novel delivery system for curcumin by using γ-CD-MOFs to increase the stability and bioavailability of curcumin. The researchers encapsulated curcumin into γ-CD-MOFs and evaluated the encapsulation efficiency (EE) and loading capacity (LC) through various γ-CD-MOF concentrations. The results showed that increasing the concentration of γ-CD-MOFs from 1 mg/mL to 3 mg/mL increased the EE from 24.77% to 67.31% and the LC from 19.83% to 30.97%, indicating the impact of γ-CD-MOF concentration on the loading efficiency of curcumin.^116^ The potential of a nasal powder formulation of CD-MOFs loaded with Eugenol (Eug), an antibacterial natural agent, was also investigated to treat bacterial rhinosinusitis. The formulation was developed employing a gas–solid adsorption method in which Eug was combined with G-CD-MoF at 90 °C. The results showed that the nasal powder effectively accumulated and maintained stability in the posterior nasal septum with a deposition distribution of 57.29–7.02.^117^

Curcumin (CUR) has significant potential for topical therapeutic applications; however, its low water solubility is a major limitation. In this study, a composite carrier system was developed by integrating γ-CD-MOFs and a β-cyclodextrin nanosponges (β-CDNS). γ-CD-MOF showed 13.9% drug loading capacity and a 267.1-fold increase in the water solubility of CUR, while β-CDNS improved drug solubility by providing additional bioadhesive properties. The resulting composite carrier (γ-CD-MOF@β-CDNS) significantly enhanced the in vitro drug release and transdermal permeation of CUR. Furthermore, its limited water absorption and excellent bioadhesive properties offer a distinct advantage for topical drug application in the treatment of exudative skin conditions. This composite carrier holds promise as a novel and effective strategy for local delivery of poorly soluble drugs.^118^

## 5 Modified CD-MOFs and Their Applications

Modified CD-MOF formulations have been developed to increase the stability of the CD-MOF structure. Several strategies, such as postmodification of cross-linked CD building blocks, modification of the surface of CD-MOFs, and the use of auxiliary molecules to enhance oxygen–metal coordination, can be employed. The modifications enhance structural stability and therapeutic efficacy by offering controlled and/or targeted drug delivery.^119–122^

In research, a chitosan (CS) modification was applied to the γ-CD-MOF structure, and CD-MOF/CS nanocapsules were prepared by an ionic gelation method in order to increase the solubility and efficiency of resveratrol (RES), which has bioavailability problems due to its low water solubility and low stability. Chitosan modification decreased the particle size from 325 to 174.6 nm, changed the zeta potential to positive. The antioxidant activity of RES-CD-MOF/CS nanocapsules increased, and in the release study, resveratrol release reached 83.9% in 24 h and exhibited a controlled release profile.^123^

Wang et al. synthesized CD-MOF nanocrystals in order to increase the solubility and oral bioavailability of indomethacin (IMC), and IMC was loaded into CD-MOFs by an impregnation method. CD-MOF@Eudragit RS microspheres were formulated using a spray drying method to take advantage of Eudragit RS, providing sustained release. The water solubility of IMC/CD-MOFs increased IMC solubility 13-fold, and in the release study, data showed that the cumulative release of IMC increased from 41% to 82% within 12 h. In vivo pharmacokinetic studies performed by the oral route revealed that the CD-MOF-based system significantly increased the bioavailability of IMC.^124^ The CD-MOF system was also investigated in combination with microneedles. CD-MOFs cross-linked diphenyl carbonate (CDF) were synthesized and loaded with quercetin (QUE) for use in the treatment of hypertrophic scars (HS). In order to increase transdermal transport, CD-MOFs were combined with soluble microneedles produced with the Bletilla striata polysaccharide (BSP). The in vivo study results concluded that the developed microneedle-CD-MOFs formulation exhibited a significant reduction in HS thickness after 21 days of treatment.^125^ A recent study also investigated the potential of CD-MOFs combined with microneedles by loading dexamethasone (DXMS) and paeonol (Pae) to enhance drug delivery for allergic rhinitis. CD-MOFs were used in the formulation to improve the solubility and stability of the drugs. The resulting microneedles were shown to effectively reach the nasal mucosa and deliver the loaded drugs.^126^

Research was conducted with the view that CD-MOFs could contribute to the development of vaccine formulations. It was aimed to investigate Sp-*g*-CD-MoF as a vaccine adjuvant to enhance the immune response against the model antigen ovalbumin (OVA). The formulation development studies involved the replacement of G-cyclodextrin metal–organic framework (G-CD-MoF) with Span 85 and encapsulation of OVA, followed by immunization of mice with the SP-G-CD-MoFOVA formulation. The results proved that the formulation induced high antigen-specific IgG titers and improved spleen cell proliferation and cell activation, demonstrating its potential as an effective vaccine adjuvant.^127^ In another research, γ-CD-based MOF structures were adopted to 2D nanosheets (NS) to evaluate the effect of particle size and morphology as a topical ophthalmic drug carrier. NS-MOFs were synthesized by the one-pot reaction of γ-CD and potassium carbonate in aqueous media. Following optimization and characterization studies, the results showed that 2D nanosheet structures were able to significantly increase the bioavailability and the retention time of dexamethasone in tear and intraocular fluids compared to 3D cubic structures and commercial product Maxidex (0.1% dexamethasone).^128^ In another study, the solubility and bioavailability of the BCS Class II drug Bazedoxifene (BZA) were enhanced using g-cyclodextrin metal–organic frameworks (MOFs). The researchers synthesized and optimized 3D CD-MOFs and 2D CD-MOFs encapsulating BZA by analyzing their drug loading capacities and pharmacokinetics. The results showed that the bioavailability of BZA loaded into 2D CD-MOF was 4.47 times higher than pure BZA and 1.38 and 4.41 times higher than BZA-3D CD-MOF and BZA-G-CD, respectively.^129^

## 6 Conclusion

3D CD-MOFs have gained significant popularity in various research fields due to their unique structural and physicochemical properties. Their applications in the drug delivery era are rapidly widening, owing to their ability to enhance solubility and biocompatibility and enable controlled drug release. These unique features are especially critical for the delivery of poorly water-soluble drugs, such as those classified under BCS Class II and IV. Moreover, the potential of 3D CD-MOFs can be further enhanced through surface modifications or combining with other drug carriers and particle size optimization, hence enabling formulation strategies tailored to specific therapeutic demands. From an industrial point of view, the manufacturing processes could be more convinient for scale-up and large-scale manufacturing compared to other nano-drug delivery systems. Based on the investigations reported to date, 3D CD-MOFs represent a versatile drug carrier for future research for a broad range of drugs, administration routes, and therapeutic applications.
